# Effectiveness of enrichment programs in developing cognitive, affective, and higher-order thinking skills among gifted students in Arab countries: a meta-analysis

**DOI:** 10.3389/fpsyg.2026.1856020

**Published:** 2026-10-19

**Authors:** Salaheldin Farah Bakhiet, Mohammad Said Soliman, Yahia Mohammed Fadul Allh Mussa, Asma Mohammed Mustafa Dafaalla, Qamrah Hatab Alsubaie

**Affiliations:** 1 Department of Special Education, College of Education, Administrative and Technical Sciences, Arabian Gulf University, Manama, Bahrain; 2 Department of Researches and Studies, Ministry of Education, Cairo, Egypt; 3 Department of Psychology, Faculty of Arts, Omdurman Islamic University, Omdurman, Sudan; 4 Department of Psychology, College of Languages and Humanities, Qassim University, Buraidah, Qassim, Saudi Arabia; 5 Department of Special Education, College of Education, King Saud University, Riyadh, Saudi Arabia

**Keywords:** affective outcomes, Arab context, cognitive outcomes, educational interventions, enrichment programs, gifted education, higher-order thinking, meta-analysis

## Abstract

**Background:**

This meta-analysis evaluated the effectiveness of enrichment programs in enhancing cognitive, affective, and higher-order thinking skills among gifted students in Arab countries, focusing on the moderating roles played by gender and grade level.

**Methods:**

Evidence was drawn from quantitative experimental and quasi-experimental research published between 2000 and 2026. Following predefined inclusion and exclusion criteria, 27 studies comprising 45 effect sizes were included in the analysis. Effect sizes were estimated with Hedges’ *g*, and a random-effects model was used to account for between-study heterogeneity.

**Results:**

The findings indicated a very large overall effect of enrichment programs on gifted students’ outcomes (*g* = 3.42). Domain-specific analyses also revealed large effects for cognitive (*g* = 2.73), affective (*g* = 5.21), and higher-order thinking skills (*g* = 3.55). Indices of heterogeneity highlighted considerable variability across studies, suggesting meaningful differences in program characteristics, measurement approaches, and research design. Moderator analyses likewise indicated that gender did not significantly influence program effectiveness across the three outcome domains. Furthermore, grade level did not significantly moderate cognitive or affective outcomes; however, it significantly moderated higher-order thinking outcomes.

**Conclusion:**

These findings indicate that enrichment programs have substantial promise for the multidimensional development of gifted learners in Arab educational contexts. However, the high heterogeneity in the results warrants caution in interpretation. More rigorous study designs, broader use of standardized assessment tools, and more fine-grained analyses of moderators are needed.

## 1 Introduction

Rapid transformations compel educational systems to invest in human capital, particularly gifted students, who represent a cornerstone of development, innovation, and creativity. In recent years, gifted education has experienced significant growth worldwide.

In the educational context, Renzulli (1977) considers giftedness and talent the result of interaction among three clusters of human traits: above-average general abilities, high levels of task commitment (motivation), and high levels of creativity. Gifted students require access to a broad, rigorous, and innovative curriculum and to enrichment programs that prepare them for future challenges and help them develop their skills. Because these students have specialized educational needs, they frequently struggle in standard classrooms (Aboud, 2026; Bakar, 2016; Wu and Yuan, 2023). Renzulli (2014) emphasizes that students actually or potentially demonstrating these traits require enriched educational environments and diverse instructional services that are typically unavailable in traditional educational programs.

The National Association for Gifted Children [NAGC], (2024) defines gifted and talented (G&T) individuals as those who perform or have the potential to perform at levels significantly above their peers in one or more domains. They require modifications to their educational experience to learn and reach their full potential.

Gifted and talented students often share the following characteristics (Kuznetsova et al., 2024):

- They come from all racial, cultural, and socioeconomic groups.
- They require adequate educational opportunities to realize their potential.
- Some may experience learning or processing disorders necessitating specialized interventions.
- They need support and guidance to develop socially and emotionally.

Recently, the concept of giftedness has undergone a fundamental shift; moving beyond a narrow definition (Golle et al., 2022), contemporary perspectives address more complex dimensions of giftedness, such as creativity, motivation, and domain-specific strengths (Sternberg and Ambrose, 2021). Gifted children are now considered a diverse group with high potential across dimensions (Mönks and Mason, 2000).

Despite this potential, gifted individuals face unique challenges, requiring targeted support. Various programs and models have been developed to foster and enhance the abilities of this population in both the cognitive and non-cognitive domains, including a wide range of enrichment programs designed to support their holistic development.

### 1.1 Enrichment programs

Growing interest in enrichment programs has led to the development of numerous models that are now widely implemented globally. These programs enhance and nurture the academic, social, creative, and intellectual skills of gifted students while addressing their needs. Most programs share a vision related to curriculum development for gifted learners, adopting a broader concept of giftedness and taking process skills into account (VanTassel-Baska and Brown, 2007).

Although the term “enrichment” is widely used, it lacks a universally agreed upon definition (Gül and Ayık, 2025). It usually refers to diverse instructional approaches beyond the standard curriculum. Enrichment programs offer gifted students the opportunity to explore topics in greater depth and complexity compared with traditional curricula (Golle et al., 2018; Reis et al., 2021; Schiever and Maker, 2003). In addition, the asynchronous development of gifted individuals indicates their need for enrichment programs. These programs help provide opportunities for talent development.

Saudi Arabia is a leading country in the Arab world in terms of educating and nurturing gifted students. The country offers various enrichment programs for students identified through approved tools, including pull-out programs in public schools and evening and weekend programs at centers for gifted students. During summer vacations, gifted student centers, universities, research centers, and major companies also organize enrichment programs (Aljughaiman, 2010, 2011a, 2011b). King Abdulaziz and His Companions Foundation collaborates with the Mawhiba Foundation to provide enrichment programs and other support initiatives to enable Saudi students to employ their intellectual abilities in generating ideas for this purpose (Subhi-Yamin, 2009). These enrichment programs develop the natural abilities of G&T students to “imagine, think forward, and establish strong connections between ideas” (p. 1,479). Two main approaches are adopted in enrichment programs for G&T students from Grade 3 to Grade 12: Renzulli’s Enrichment Triad Model (ETM) and the Cognitive Research Trust (CoRT) program developed by Edward de Bono using a range of differentiation strategies.

The Renzulli ETM is used in withdrawal programs for G&T students; students are generally withdrawn from classes once a week for the program (Renzulli, 1987). Renzulli’s model is designed to encourage creative productivity by exposing G&T students to diverse topics by providing informational content and by allowing students to self-select areas of interest that will be the focus of their learning (Lyons and Reis, 2013). Enrichment is embedded in exposing G&T students to content that they do not usually learn in regular classrooms, thereby promoting students’ thinking and communication development and providing them with opportunities to apply their knowledge and creative ideas (Lyons and Reis, 2013). In Renzulli’s school enrichment model, schools offer enrichment for gifted students in resource rooms in a school building. G&T students undertake independent learning here or study with a resource teacher (Subhi-Yamin, 2009).

The CoRT thinking program has been used in gifted education and enrichment programs in Saudi Arabia, including in the Mawhiba summer programs organized by the King Abdulaziz and His Companions Foundation for Giftedness and Creativity (Almakhalid, 2012). The contemporary approach to educating G&T students in Saudi Arabia uses differentiated lessons in regular classroom settings, providing additional curriculum resources and materials. CoRT’s model focuses on developing and using teaching and learning materials to directly teach thinking as a fundamental and essential skill (de Bono, 2009). CoRT’s thinking skills courses provide students with applied learning experiences to promote independent thinking (de Bono, 2009). In the CoRT program, advanced educational materials, additional research projects, small-group learning, flexible educational programs, and reading clubs are used; furthermore, curricula are made compact, mentors are involved, research opportunities are expanded, and teaching is aimed at developing higher-order thinking skills (Subhi-Yamin, 2009).

In addition to Saudi Arabia, many Arab countries have shown interest in gifted students. The early 1980s, specifically 1982, marked the beginning of efforts to provide services for gifted students in Jordan, with the opening of the Al-Salt Center for the Gifted and Talented. During the 1986–1987 academic year, Ministerial Decision No. 135-86 was issued, officially authorizing the provision of services for gifted students across Jordanian schools (Ghanem, 2011). At that time, the Jordanian state formally adopted a national project for gifted education. This initiative included identifying gifted students in schools using intelligence tests, offering enrichment programs for third- and fourth-grade students, and monitoring academic progress in Arabic, mathematics, and science (Al-Hroub, 2023). In 1995, the Directorate of Special Education and the Gifted Programs Division were established. These developments were preceded by the founding of the Jubilee School in 1977 as a community initiative The school began operations in the 1993–1994 academic year as the first specialized school for gifted and high-achieving students. It is a nongovernmental, non-profit secondary school (Ghanem, 2010; Al-Shobaki, 2007). Over the past four decades, gifted education in Jordan has expanded significantly over the past four decades, with the number of gifted care centers rising to 19 across the country’s governorates. These centers offer after-school enrichment programs for students at the basic and upper stages, providing several hours of weekly instruction in subjects such as science, mathematics, Arabic, English, computer skills, music, and art. Additionally, the King Abdullah II Schools for Excellence were launched, beginning with a school in Zarqa in the 2001–2002 academic year and expanding to 13 schools by the 2012–2013 academic year (Al-Hroub, 2023). Statistics from the Jordanian Ministry of Education for 2021 indicate the availability of 75 schools offering resource room services for gifted students, benefiting 1,300 students annually. Moreover, 1,500 students from schools across Jordan are enrolled in academic acceleration programs, with all necessary financial, technical, and administrative support provided (Jordanian Ministry of Education, 2021; Al-Rababa’a, 2023).

Official attention to gifted education in the United Arab Emirates began with the “School for All” initiative, which was part of the Ministry’s 2010–2020 strategy and aimed to integrate special education services, including those for gifted students, into public and private schools. In 2008, the country launched an initiative to develop the skills of gifted and talented students. Federal Law No. 29 of 2006 was enacted, mandating the provision of special educational services for gifted students. This was followed in 2010 by the issuance of the “General Rules for the Provision of Special Education Programs and Services,” which outlined procedures for identification, in-school support, and advanced Individualized Education Plans (IEPs) for gifted students (Alhosani et al., 2022). At the emirate level, institutions such as the Abu Dhabi Education Council (ADEC, 2011) and Dubai’s Knowledge and Human Development Authority (KHDA, 2011) played pivotal roles in supporting programs and regulating private schools (Sharma, 2019). The last decade has witnessed significant qualitative progress in the care of gifted individuals in the UAE, driven by the nation’s vision for a knowledge-based economy and innovation. In 2014, the Ministry adopted an “Integrated System for Identifying and Nurturing the Gifted” (AlGhawi, 2017). Initiatives expanded to include nongovernmental entities such as the Hamdan bin Rashid Al Maktoum Foundation for Distinguished Academic Performance and the Emirates Association for the Gifted. A key project is the Hamdan Center for Giftedness and Innovation, established in 2015, which aims to identify and support gifted students in STEM fields through specialized training programs and local and international competitions. Since the launch of the student gifted identification program in 2017, 39,000 students across public and private schools have been assessed, resulting in the identification of 2,207 gifted students from 122 schools. The program aims to reach a total of 5,000 gifted students by 2030. Furthermore, the scope of these programs expanded from 10 schools in 2008 to 250 schools in the following years. The UAE Ministry of Education’s policies emphasize linking gifted programs with innovation laboratories and scientific mentorship, as well as participation in global programs, such as the Cambridge Pre-University curriculum, to support the UAE’s vision of preparing future leaders (Alhosani et al., 2022).

Egypt’s efforts to educate the gifted represent one of the earliest such initiatives in the Arab world, dating back to the early 19th century, when Muhammad Ali Pasha, the then-ruler of Egypt, sent gifted and talented individuals to Europe. These efforts evolved into a formal institutional framework in 1932, when the Ministry of Education established special classes attached to the Teacher Training Institute. These classes were later transformed into a model school based on the project-based learning method. In 1962, a ministerial decree established Egypt’s first official school for gifted students, the Ain Shams School for the Gifted (Ayoub et al., 2022). In 2011, the Ministry of Education launched STEM schools, specialized institutions for high achievers in science and mathematics, aimed at providing an educational environment grounded in research and problem-solving. To date, there are 23 such schools across 18 governorates, serving 6,000 gifted students (Al-Etrabiy, 2019). Furthermore, the Ministry of Higher Education has established numerous specialized centers within universities to support and nurture gifted students.

In Sudan, gifted education has evolved from informal practices to more systematic approaches to identification and specialized educational provision. Early practices resembling academic acceleration can be traced to traditional Quranic schools (Khalwa), where learners progressed according to their individual pace. More organized provision later emerged through model secondary schools for high-achieving students and the establishment of the Sudanese Association for Gifted Children at the University of Khartoum in the late 1980s. A major shift toward formal gifted education occurred in the early 2000s, culminating in the establishment of specialized schools for gifted students in Khartoum, Khartoum North, and Omdurman in 2005. Identification relied on multiple criteria, including academic achievement, intelligence, creativity, and teacher nominations, while educational provision incorporated curriculum enrichment, thinking-skills development, psychological guidance, and extracurricular activities. Institutional support was further strengthened in 2006 with the establishment of the National Authority for the Nurture of Gifted Children under Presidential Decree No. 221, followed by the expansion of specialized schools in other states, including Gezira, Gedaref, and River Nile. Despite this progress, gifted education in Sudan has continued to face challenges related to funding and infrastructure, specialized teacher preparation, legislative support, and geographical availability of services (Bakhiet and Mohamed, 2022).

Several studies in the Arab world have evaluated the effectiveness of enrichment programs from multiple perspectives (Al-Aqil et al., 2019; Al-Qarni, 2010; Aldaoud, 2010; Aljughaiman, 2011b, 2011c; AlQahtani, 2009; Maajini, 2006). Research has identified numerous gifted education programs in Saudi Arabia. Most reports examine variables directly related to the quality of enrichment programs, including instructor qualifications, student performance, and the attitudes of both teachers and students (Alfaiz et al., 2022).

In recent decades, several reputable enrichment models have been developed, including Renzulli’s (1977) ETM, which later evolved into the Schoolwide Enrichment Model (Renzulli and Reis, 1985), and VanTassel-Baska’s (1995) Integrated Curriculum Model. Evidence-based programs supported by experimental data are necessary to demonstrate the effectiveness and scientific value of such programs to the stakeholders of gifted education, including teachers and policymakers (Eysink et al., 2020; Golle et al., 2018; Kim, 2016).

Program evaluation is fundamental to developing high-quality educational programs. It allows decision-makers to understand the extent to which a program is achieving its aims, how these aims are being achieved, what obstacles hinder progress, and whether the initiative deserves continued support and funding (Chen and Chen, 2020). For program designers, evaluation provides reliable evidence for assessing program effectiveness. For students and parents, it allows them to assess program quality and offer suggestions for improvement.

Evaluation of gifted education programs is a key component of the program cycle (Callahan et al., 2017). Through evaluation, best practices are identified for students who align with the dynamic and evolving definition of giftedness (Chen and Chen, 2020). Effective evaluation of gifted education programs encompasses not only product but also process outcomes. While the former reveals the overall effectiveness of the program, the latter focuses on the extent to which the program was implemented in relation to design (Callahan et al., 2017).

Meta-analyses are widely employed as they draw on a range of individual studies to obtain a composite conclusion (Glass, 1976) and clarify why findings across studies vary. This research method can guide researchers and policymakers in developing future policies (Ogurlu, 2020; Steblay et al., 2011).

### 1.2 Contributions of meta-analyses to research on gifted education

Steenbergen-Hu and Olszewski-Kubilius (2016) and Vaughn et al. (1991) hold that meta-analyses make unique contributions to research on gifted education. Their results are based on systematic and reproducible steps and are therefore reliable. By summarizing evidence in the literature, broader insights can be obtained. Meta-analyses enable researchers to examine multiple independent variables and potential moderating effects.

Numerous studies have been conducted on enrichment programs in gifted education across many Arab countries. The results of these individual studies vary and may be contradictory. Furthermore, meta-analyses that examine the effects of interventions within the Arab context are scarce. A literature review found only one meta-analysis on enrichment programs in gifted education, which investigated overall effect sizes across a wide range of cognitive, emotional, and reasoning skills (Aljughaiman and Ayoub, 2013).

Gül and Ayık (2025) note that it is essential to systematically evaluate the effectiveness of enrichment programs. Notably, studies published between 2014 and 2024 reveal considerable methodological variation as different definitions of enrichment are used and outcome measures in many quantitative studies often rely on unvalidated tools and small-scale research designs.

### 1.3 Cognitive skills

Gifted students need cognitive skills to realize their potential (Reis et al., 2021). Kaplan Sayı and Yurtseven (2022) identified several core learning skills that gifted learners must have, including listening and observation, perception, reading comprehension, note-taking, summarization, data analysis and organization, and effective communication.

Reis et al. (2021) added the need for essential learning skills, such as planning, resource utilization, organization, time management, decision-making, and self-assessment. These skills allow gifted students to become independent. Macleod and Hayden (2016) emphasize that these skills are critical and support long-term academic achievement by enhancing adaptability. By focusing on developing cognitive skills, enrichment programs can contribute meaningfully to gifted students’ educational experience.

### 1.4 Affective skills

While gifted individuals possess exceptional cognitive abilities, such individuals, similar to their nongifted peers, also experience emotional, social, and interpersonal challenges (Garland and Zigler, 1999; Piirto, 1992), which could stem from their asynchronous patterns of development (Morawska and Sanders, 2008; Neihart, 1999). Researchers suggest that, because gifted individuals are out of sync with their peers, they may feel incompatible with their nongifted counterparts, leading to greater personal difficulties (Guénolé et al., 2013; Liratni and Pry, 2011; Webb et al., 2016). This indicates the need for gifted individuals to develop their socioemotional skills, a goal of many enrichment programs. Enrichment programs can contribute to the development of socioemotional skills by fostering positive self-concept, self-efficacy, self-management, motivation, emotional resilience, and independence (Gül and Ayık, 2025). Subotnik et al. (2011) emphasize that socioemotional factors play a vital role in allowing gifted individuals to reach their full potential. This aligns with Gül and Ayık’s (2025) study, which asserted that supporting the socioemotional needs of gifted individuals can contribute to their overall academic and personal development.

### 1.5 Higher-order thinking skills

Higher-order thinking skills (HOTS) occupy a central position among the cognitive abilities associated with giftedness (Greiff et al., 2015). These skills require more than using routine cognitive processes to critically analyze information, integrate diverse perspectives, and construct new understandings that transcend disciplinary boundaries (Subotnik et al., 2023). Higher-order thinking focuses on processing information in complex ways to generate novel ideas. These skills are fundamentally based on Bloom’s Taxonomy, including analysis, evaluation, and innovation. Most enrichment programs foster HOTS among gifted students across a range of educational contexts. Olenchak and Renzulli (1989) emphasized that enrichment programs have a pivotal role to play in promoting advanced levels of creativity and creative problem-solving. The World Economic Forum (2018) found that major global companies predict that creativity, critical thinking, and complex problem-solving are among the most important skills for employers. These growing demands lead educational systems, including those in Arab countries, to introduce enrichment programs that are designed to develop these competencies in gifted students. Despite the growing application of such programs, the accumulated evidence for their effectiveness across cognitive, affective, and higher-order thinking domains in Arab educational contexts remains insufficiently synthesized.

### 1.6 Literature review

A 2013 meta-analysis by Aljughaiman and Ayoub evaluated the effects of enrichment programs in the Arab world based on the Oasis Enrichment Model (OEM) on various dimensions of gifted education in Saudi Arabia between 2009 and 2011 (Aljughaiman and Ayoub, 2013). A total of 35 studies were included in this meta-analysis. Enrichment programs had statistically significant positive effects on analytical abilities, creative abilities, thinking skills, critical thinking, future problem-solving, attitudes toward learning, motivation, decision-making, content of knowledge, classroom performance, and personal and social traits, and there were no significant effects on integrated science processes.

Alzahrani (2023) reviewed the Saudi model of enrichment in published studies on the OEM during the period from 2004 to 2022, selecting 60 studies. This investigation concluded that the OEM for gifted students, developed by Aljughaiman, revolutionized giftedness, gifted education, and gifted student care. Enrichment programs based on the OEM demonstrate tangible positive effects, providing researchers in the Arab world with a number of studies to illustrate the impact of these programs on gifted students. Alzahrani (2023) demonstrated that the OEM had a significant impact on learning environments. The 20-year application of this model led to the development of cognitive and emotional skills and HOTS, including creative thinking, innovative problem-solving, critical thinking, and future-oriented thinking.

Two international meta-analytical studies have investigated the effects of enrichment on gifted education (Gül and Ayık, 2025; Kim, 2016). These show average effect sizes ranging from small and medium to large. Kim (2016) provided a large mean effect size of enrichment programs on academic outcomes (*g* = 0.96) and a medium mean effect size for socioemotional outcomes (*g* = 0.55). The results likewise showed that the mean effect size varied across grade levels: it was medium in elementary grades, large in middle grades, and very large in upper secondary grades for academic outcomes. The average effect size for socioemotional outcomes also varied with grade level: very large in middle grades, medium in elementary grades, and small in upper secondary grades. Gül and Ayık (2025) found a large mean effect size of enrichment programs on cognitive skills (*g* = 1.14), a medium average effect size for interpersonal and emotional skills (*g* = 0.51), and a small average effect size for socioemotional skills (*g* = 0.44) in 16 studies published between 2014 and 2024. They also found that the average effect size varied with grade level, being large across all grades (elementary, middle, and high school) for cognitive skills (*g* = 1.34, 1.10, and 1.14, respectively). Conversely, small effects were observed in elementary schools, medium effects in middle schools, and medium to large effects in high schools (*g* = 0.21, 0.54, and 1.07, respectively) for emotional skills.

Young et al. (2017) investigated the effects of out-of-school time on student interest in STEM in 15 studies conducted in the United States from 2009 to 2015. The results suggested that out-of-school time had a positive effect on student interest in STEM (*g* = 0.37, 95%CI [−0.54, 2.50], using a random-effects model).

A meta-analysis of 16 STEM summer bridge programs for university students was conducted by Bradford et al. (2021). The results showed that program participation had a medium-sized effect on first-year overall GPA (*d* = 0.34, 95%CI = [0.16, 0.52]) and first-year university retention (odds ratio [OR] = 1.747). A meta-analysis by Lynch et al. (2023) examined 37 studies of summer programs in mathematics for children in pre-K-12 to determine the resources and characteristics that predict stronger student achievement. The study indicated an average weighted impact estimate of +0.10 standard deviations on mathematics achievement outcomes.

Despite the generally positive findings reported in previous meta-analyses, this study addresses an important gap in the gifted education literature by providing an updated and multidimensional meta-analytic synthesis of enrichment programs for gifted students across Arab educational contexts. Existing syntheses have either focused on specific enrichment models or national contexts, particularly Saudi Arabia (Aljughaiman and Ayoub, 2013; Alzahrani, 2023), or have examined enrichment programs across broader international contexts (Gül and Ayık, 2025; Kim, 2016), limiting their ability to offer a comprehensive assessment of enrichment practices across Arab educational settings. Moreover, previous findings vary in the magnitude of reported effects across cognitive, affective, and higher-order thinking outcomes, as well as across student and program characteristics. This gap is particularly important given the diversity of enrichment approaches implemented in Arab countries and the potential influence of contextual and methodological factors on their effectiveness. Therefore, a comprehensive meta-analysis is necessary to synthesize the available evidence from Arab countries; estimate the overall effectiveness of enrichment programs across cognitive, affective, and higher-order thinking outcomes; and examine whether their effects vary according to relevant study, participant, and program characteristics.

### 1.7 Research questions

This meta-analysis examined the effectiveness of enrichment programs in developing cognitive skills, affective skills, and higher-order thinking skills (HOTS) among gifted students in Arab countries. Accordingly, the study was specifically limited to enrichment interventions implemented with gifted students in Arab educational contexts and examined in studies that met the predefined eligibility criteria. The study addressed the following research questions:

1. What is the overall effect of enrichment programs on the cognitive skills, affective skills, and HOTS of gifted students in Arab countries?
2. To what extent do grade level and gender moderate the effects of enrichment programs on these outcomes among gifted students in Arab countries?

## 2 Materials and methods

### 2.1 Selection of studies

To ensure comprehensive coverage of empirical studies on this topic, publications from 2000 to 2026 were searched using the following keywords and phrases: “gifted” OR (talented, high ability, high-IQ, academically able, advanced students, highly able, exceptional student). We also used the following search term: “enrichment-program” OR (pull-out program, summer program, Saturday program, extracurricular program). The study employed various combinations of the two sets, pairing one from the first with one from the second, such as (1) gifted AND enrichment-program, (2) talented AND pull-out program, and (3) gifted AND summer program. The keywords were searched in English, along with their Arabic equivalents. The authors reviewed the following digital databases: Dar Almandumah, Shamaa, ERIC, Arab PsycINFO, OATD, PROQUEST, SAUDI DIGITAL LIBRARY, and Arab World Research Source. The following Arabic journals in the field of gifted education and special education were manually reviewed using their official websites: (1) *The International Journal of Talent Development* (ISSN: 2415–4,563) from 2013 to the present, (2) *Special Education and Rehabilitation* (ISSN: 2314–8,608) from 2013 to the present, and (3) *Arab Journal of Giftedness and Disabilities Sciences* (ISSN: 2537–0480) from 2017 to the present. In addition, four international journals were manually searched using their official websites: (1) *Gifted Child Quarterly*, (2) *Gifted and Talented International*, (3) *Roeper Review*, and (4) *High Ability Studies*.

A more extensive search was conducted using the following set of procedures: (1) searching for abstracts on digital databases; (2) searching for new studies using Google and Google Scholar; (3) searching for the publication records of Arab researchers who have published more than one article on enrichment programs for the gifted; (4) personal correspondence with researchers working in special education, gifted education, and educational psychology departments at Arab universities; and (5) searching the reference lists of the retrieved studies to find additional publications.

After duplicates were removed, the search yielded 88 published and unpublished records. The selected works included peer-reviewed articles, theses, dissertations, reviews, books, book chapters, and conference papers.

### 2.2 Study inclusion and exclusion criteria

Investigation was conducted according to the following inclusion and exclusion criteria.

(1) Study Topic: The study topic must address enrichment programs for gifted students in a K-12 setting.

(2) Language: Only studies that were published in Arabic or English were included.

(3) Publication Type: To avoid publication bias (Rosenthal, 1991), published and unpublished works including theses, dissertations, reviews, books, book chapters, and conference papers were included.

(4) Publication Year: Studies published between 2000 and 2026 were selected.

(5) Appropriate Research Methods: Quantitative studies and those with experimental or quasi-experimental designs, such as pre- and post-tests, or studies with experimental and control groups, or those with only experimental groups were included; qualitative studies, theoretical reviews, and case studies were excluded.

(6) Statistical information/data: The studies included sufficient statistical information to calculate effect sizes (Hedges’ *g*), such as means, standard deviations, *p*-values, t-values, or *F*-values, ANOVA tables, and sample size.

The inclusion and exclusion criteria were discussed and agreed upon by all authors (94% agreement). After the above criteria were applied, the final sample included 27 studies (encompassing 45 effect sizes). Figure 1 presents a flowchart of the selected studies.

**Figure 1:**
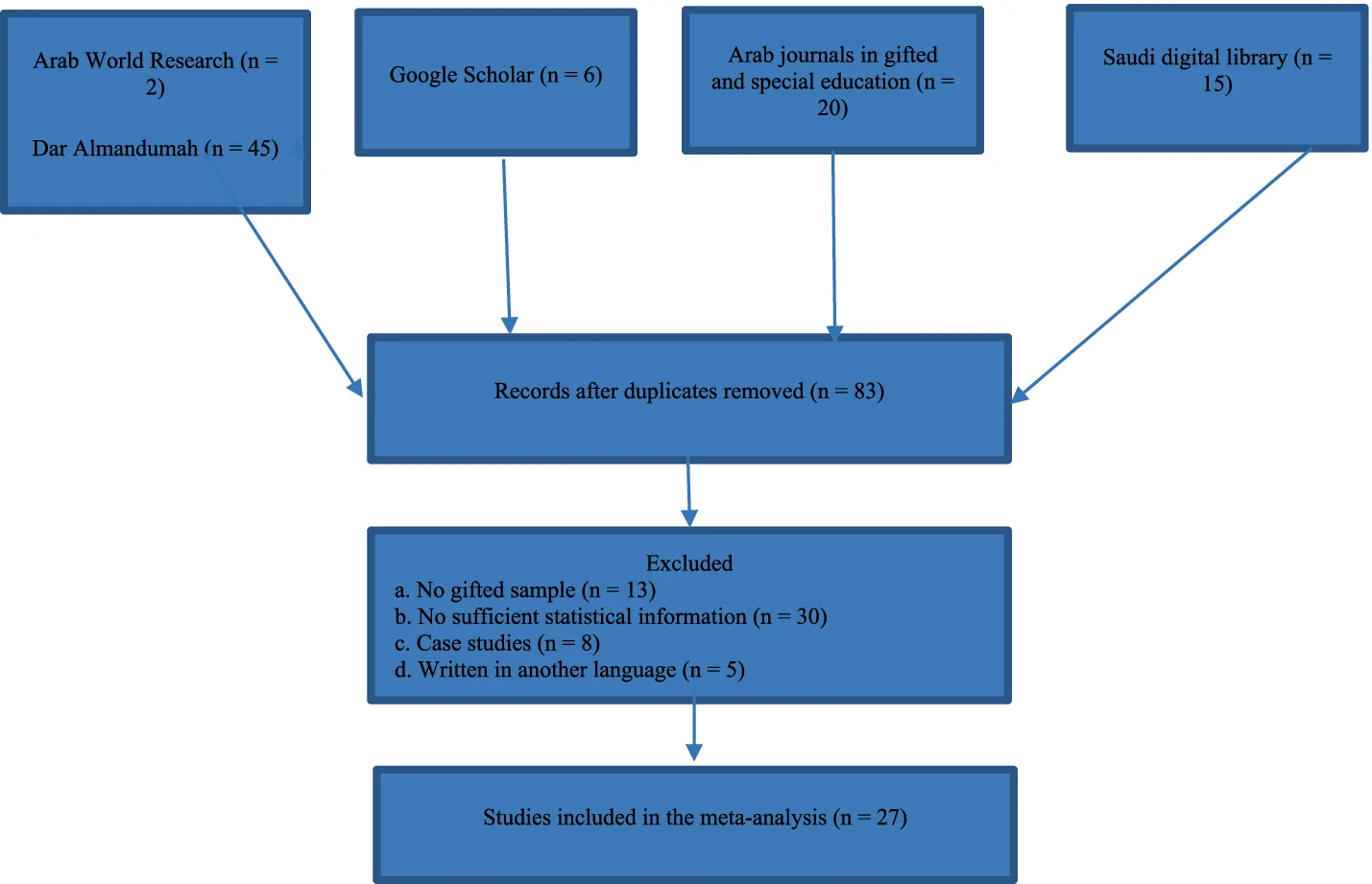
Flow diagram of study selection.

### 2.3 Study coding

Pilot coding was conducted with five studies, producing rigorous reviews. The final coding included (A) study overview and (B) study data. The first dimension included the following information: (1) Study authors; (2) Year of publication; (3) Article type (journal article, thesis, dissertation, or conference paper); (4) Sample size; (5) Design type (experimental or quasi-experimental); and (6) Study moderating factors, classified as (a) Gender (male, female, or combined) and (b) Grade level or age (preschool, elementary [K-6], middle school [7–9], or high school [10–12]). The second dimension included the following information: (a) Effect size for each study; (b) Standard error for each effect size; (c) Lower and upper CI; and (d) *P*-value.

The outcome measures were categorized into cognitive, affective, and higher-order thinking outcomes. In each, a wide range of knowledge and skills were measured. The cognitive outcomes included academic achievement, performance in acquiring scientific and mathematical concepts, language expression skills, and cognitive skills. The affective outcomes included self-regulation, motivation, self-concept, self-efficacy, self-esteem, and attitudes toward specific topics. Creative thinking, critical thinking, creative problem-solving, problem-solving skills, and decision-making skills were considered higher-order thinking outcomes.

A coding protocol was developed, and the first two authors worked independently. Inter-rater reliability was 0.91. Where difficulties arose during the course of coding, the authors resolved discrepancies via discussion.

### 2.4 Effect size calculation

Effect size here indicates the strength and effectiveness of an enrichment program. Lipsey and Wilson (2001) noted that effect sizes are biased where they are based on small sample sizes, particularly those with fewer than 20 participants. The samples in the studies in this meta-analysis were often small; therefore, Hedges’ *g*-index was chosen as the effect size indicator to eliminate sample size bias (Borenstein, 2009; Glass et al., 1981; Hedges and Olkin, 1985). The effect sizes of the research outcomes that were included in the studies were calculated using the raw means, standard deviations, and sample sizes of the studies. If the comparison involved a single sample that underwent pre- and post-testing, the d-index was calculated first, and then it was converted to Hedges’ *g*-index (Rosenthal, 1991). Cohen’s (1988) criteria were used to interpret standardized mean differences, and the effect sizes were summarized as small (0.20), medium (0.5), and large (0.80). Positive effect sizes were interpreted to indicate that the enrichment-program groups had stronger results compared with the control groups.

### 2.5 Heterogeneity test

In a meta-analysis, each study has its own unique characteristics and results, and the differences between the results of these retrieved studies are the group’s heterogeneity. It is crucial to evaluate and address these differences in meta-analyses (Baker et al., 2009). To achieve this, researchers typically use the Q test and the *I*^2^ statistic to assess heterogeneity. The Q test is a statistical tool used to determine whether there are significant differences in the results of each study in the meta-analysis. If the *p*-value is greater than 0.1, the results are similar, and a fixed-effects model can be adopted for the meta-analysis. However, if the *p*-value is less than or equal to 0.1, this indicates significant differences between the studies, and, in this case, a random-effects model should be used for the meta-analysis (Borenstein et al., 2010). However, the I^2^ statistic is used to assess the relative magnitude of the variance between studies. Its value ranges from 0 to 100, where higher values indicate greater variance. Generally, when *I*^2^ is less than 25%, the variance between studies is low; when *I*^2^ is between 25 and 50%, the variance is moderate; and when *I*^2^ is greater than 75%, the variance is high (Higgins et al., 2003).

### 2.6 Moderator analysis

Moderator analyses were performed to determine whether the magnitude of the effects of enrichment programs varied by certain study characteristics. Two categorical moderators specified during the coding process were examined: gender (male, female, or combined samples) and grade level (kindergarten/preschool, elementary, middle, and/or secondary school). Moderator analyses were conducted separately for cognitive, affective, and higher-order thinking outcomes. Differences in pooled effect sizes across moderator categories were evaluated with the between-group heterogeneity statistic (Qb). A statistically significant Qb value indicated that effect sizes differed significantly across categories of the moderator. All subgroup estimates were calculated using a random-effects model owing to substantial between-study heterogeneity.

### 2.7 Publication bias test

Publication bias is defined as the relationship between the likelihood that a study is published on a particular topic and its effect size. It can occur when researchers refrain from publishing for extraneous reasons. A high probability of publication bias in meta-analyses can negatively impact the accuracy of the pooled effect size (Van Aert et al., 2019), leading to a misreported average effect size (Borenstein, 2009). For this reason, the probability of publication bias in the studies included in this paper was tested before the effect sizes were determined.

A funnel plot was used to assess whether the measured effect size was related to its SE. An asymmetric funnel plot may indicate a small-study effect size, suggesting that the scientific literature is susceptible to publication bias (Sterne et al., 2000). Duval and Tweedie’s (2000) trim and fill methods are non-parametric methods that rely on the symmetry of the funnel plot to estimate the number of missing studies that should be added to the meta-analysis to obtain a symmetric funnel plot. Egger’s regression test predicts a statistically significant correlation between effect size and standard error, indicating that smaller studies reported significantly stronger effects (Egger et al., 1997). Finally, the fail-safe N indicates the number of studies needed to make the overall effect statistically non-significant (Sutton, 2005).

## 3 Results

### 3.1 Overall effect size and heterogeneity

The relationship between enrichment programs and the overall effect of student skills is presented in Table 1. Regarding evaluation criteria, according to Cohen (1988), the combined effect size of enrichment studies on students’ skills (*g* = 3.42, 95% CI: 2.54, 4.32, *Z* = 7.5835, *p* < 0.001) was greater than 0.80, indicating that enrichment studies had a large effect on gifted students’ skills.

**Table 1:** Effect sizes and heterogeneity tests of enrichment studies (random-effects model).

Statistics	Values
K	27
NES	45
Hedges’ *g* [CI (95%)]	3.43
Tau-squared	8.84
*P*	0.000
*z*-value	7.58
Classic fail-safe-N	32,863
Trim and fill	18
Heterogeneity	
Qtotal	1135.65
*p*-value	0.0001
Df	44
*I* ^2^	98.79%

The *Q* test outcomes indicated noteworthy variation in terms of effect sizes among studies (*Q* = 1,135.65; df = 44, *p* < 0.001). Additionally, the *I*^2^ statistic was determined to be 98.79, which signified substantial heterogeneity in effect sizes; specifically, approximately 98.79% of the observed variance discrepancy can be attributed to a genuine effect instead of sampling error.

The random-effects model was used to pool effect sizes and address sample heterogeneity. The random-effects model in meta-analysis assumes that each study’s true effect varies around an overall mean μ rather than being identical. In all, 45 effect sizes were extracted from 20 studies. Figure 2 displays the effect sizes, while Table 2 summarizes the articles included in the current meta-analysis.

**Figure 2:**
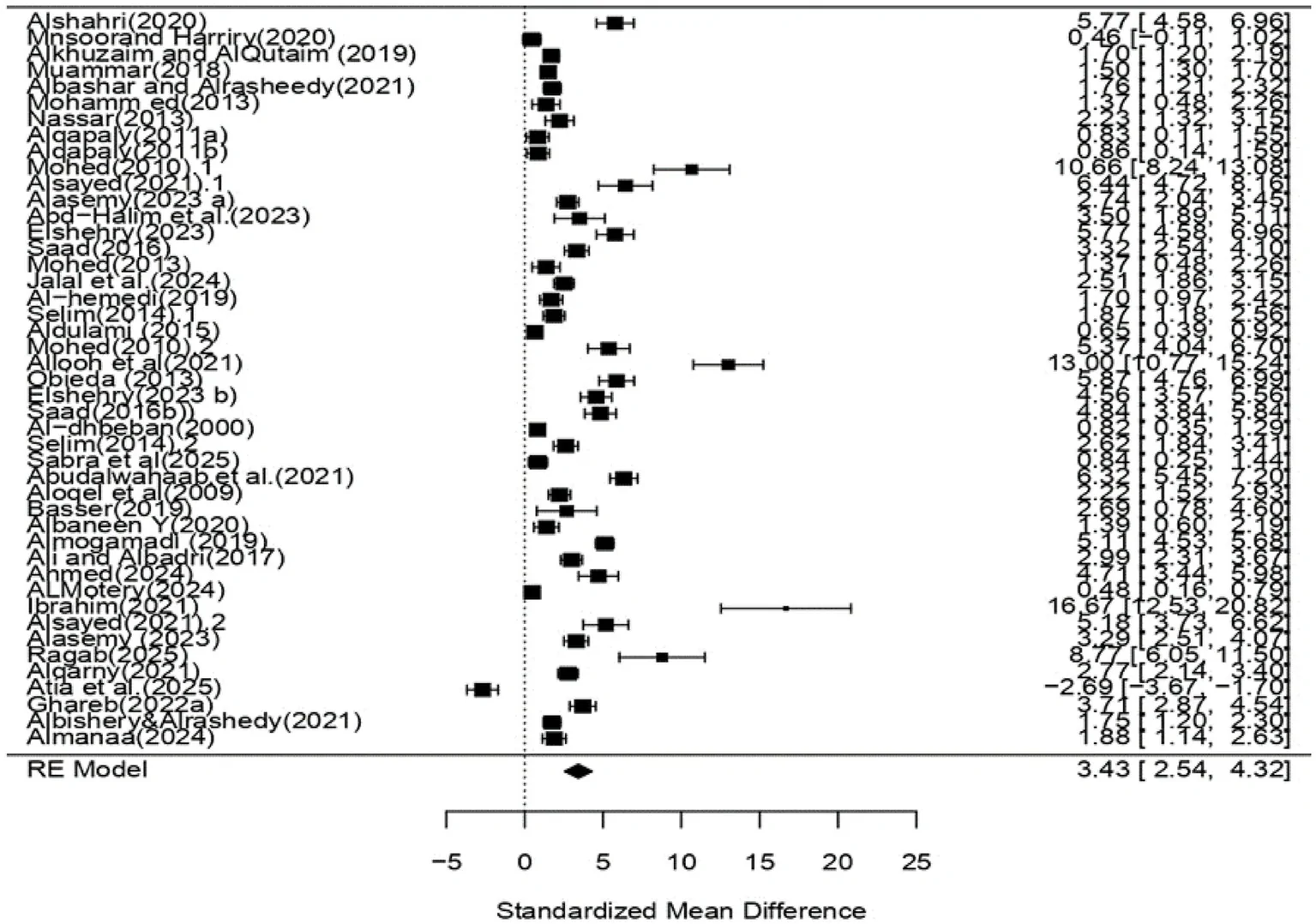
Forest plot of effect sizes using a random-effects model.

**Table 2:** Articles included in this study’s meta-analysis.

Authors	Type of comparison	Participants (N)	Type of Publication	Grade level	Gender	Nationality
Mnsoor and Harriry (2020)	Exp-Cont	29/21	Article	Secondary	Female	K. S. A
Alkhuzaim and Al-Qutaim (2022)	Pre-post	42	Article	Secondary	Female	K. S. A
Muammar (2022)	Pre-post	125	Article	Secondary	M&F	K. S. A
Al-Bishr (2021)	Exp-Cont	35/35	Article	Primary	Male	Egypt
Mohammad (2013)	Pre-post	21	Article	Kindergarten	M&F	Egypt
Nassar (2013)	Exp-Cont	15/15	Article	Primary	Female	Bahrain
Alqapaly and Grwan (2009)	Exp-Cont	16/16	Dissertation	Middle	Male	K. S. A
Alqapaly and Grwan (2009)	Exp-Cont	16/16	Dissertation	Middle	Male	K. S. A
Mohamed (2013)	Exp-Cont	20/20	Conference paper	Primary	M&F	K. S. A
Mohamed (2013)	Exp-Cont	20/20	Conference paper	Primary	M&F	K. S. A
Al-Louh et al. (2021)	Pre-post	34	Article	Secondary	Female	Palestine
Asimi (2023)	Exp-Cont	30/30	Article	Secondary	Male	Yemen
Asimi (2023)	Exp-Cont	30/30	Article	Secondary	Male	Yemen
Obaida (2013)	Pre-post	33	Article	Secondary	Male	K. S. A
Al-Qarni (2021)	Pre-post	38	Article	Secondary	Male	K. S. A
Almania (2024)	Pre-post	20	Article	Secondary	Female	K. S. A
Alshahri (2023)	Pre-post	28	Article	Middle	Male	K. S. A
Saad (2016)	Exp-Cont	30/30	Article	Middle	M&F	Egypt
Sabra et al. (2025)	Exp-Cont	24/23	Article	Middle	M&F	Syria
Jalal et al. (2024)	Pre-post	33	Article	Primary	Male	Egypt
Atia (2025)	Exp-Cont	15/15	Article	Secondary	M&F	Egypt
Gharib (2022)	Exp-Cont	30/30	Article	Secondary	M&F	Egypt
Al-Dhabiban (2000)	Exp-Cont	49/31	Article	Middle	Male	K. S. A
Al-Hemedi (2019)	Pre-post	20	Article	Primary	Female	K. S. A
Jamali (2019)	Pre-post	15	Article	Primary	Female	Bahrain
Selim (2014)	Exp-Cont	22/25	Article	Kindergarten	M&F	K. S. A
Basser (2019)	Pre-post	4	Article	Kindergarten	Male	K. S. A
Al-Dulaimi (2015)	Pre-post	115	Article	Secondary	Male	K. S. A
Abdulhalim et al. (2023)	Exp-Cont	8/7	Article	Kindergarten	M&F	Yemen
Al-Sayed et al. (2021)	Pre-post	16	Article	Kindergarten	M&F	Egypt

To address the first research question, the following sections illustrate the effects of enrichment programs on the cognitive and affective abilities and HOTS of gifted students.

### 3.2 Maximizing cognitive skills

The random-effects model indicated that the 20 effect sizes ranged from 0.46 to 10.65. A large effect was found on the mean effect size (*g* = 2.73, 95% CI [1.73, 3.72]), which indicated that enrichment interventions had a large effect on cognitive skills (Table 3). The *Q*-value was determined to be 297.03 (df = 19; *p* < 0.001), refuting the null hypothesis positing identical true effect sizes. Moreover, the *I*^2^ statistic of 98.24 signifies substantial heterogeneity in terms of effect sizes among the studies. This statistic suggests that approximately 98.24% of the observed variance in effect sizes is attributable to genuine differences rather than sampling errors.

**Table 3:** Effectiveness of enrichment programs on cognitive skill development.

N	Study	Effect sizes	SE	Lower CI 95%	Upper CI 95%
1	Alshahri (2023)	5.76	0.369	4.58	6.96
2	Mnsoor and Harriry (2020)	0.46	0.084	−0.11	1.02
3	Alkhuzaim and Al-Qutaim (2022)	1.70	0.065	1.20	2.19
4	Muammar (2022)	1.50	0.011	1.30	1.70
5	Al-Bishr (2021)	1.76	0.079	1.21	2.32
6	Mohammad (2013)	1.37	0.206	0.48	2.26
7	Nassar (2013)	2.23	0.217	1.32	3.15
8	Alqapaly and Grwan (2009)	0.83	0.136	0.11	1.55
9	Alqapaly and Grwan (2009)	0.86	0.137	0.14	1.59
10	Mohammed (2010)	10.66	1.520	8.24	13.08
11	Al-Sayed et al. (2021)	6.44	0.773	4.72	8.16
12	Asimi (2023)	2.75	0.129	2.04	3.45
13	Abdulhalim et al. (2023)	3.50	0.676	1.89	5.11
14	Alshahri (2023)	5.77	0.368	4.58	6.96
15	Saad (2016)	3.32	0.159	2.54	4.10
16	Mohammad (2013)	1.37	0.206	0.48	2.26
17	Jalal et al. (2024)	2.51	0.108	1.86	3.15
18	Al-Hemedi (2019)	1.70	0.136	0.97	2.42
19	Selim (2014)	1.87	0.123	1.18	2.56
20	Al-Dulaimi (2015)	0.65	0.018	0.39	0.92

### 3.3 Developing affective skills

Seven effect sizes were extracted to describe the impact of enrichment studies on the affective skills of gifted students. These ranged from 0.82 to 13.00. A large effect was found on the mean effect size (*g* = 5.21, 95% CI [2.47, 7.94]), indicating that enrichment interventions had a large effect on affective skills (Table 4).

**Table 4:** Effectiveness of enrichment programs on affective skill development.

N	Study	Effect sizes	SE	Lower CI 95%	Upper CI 95%
1	Mohammed (2010)	5.73	0.46	4.04	6.70
2	Al-Louh et al. (2021)	13.,01	1.30	10.77	15.24
3	Obaida (2013)	5.87	0.32	4.76	6.99
4	Alshahri (2023)	4.56	0.26	3.57	5.56
5	Saad (2016)	4.84	3.8444	3.84	5.84
6	Al-Dhabiban (2000)	0.82	0.06	0.35	1.29
7	Selim (2014)	2.63	0.16	1.84	3.41

Heterogeneity analysis presented statistically significant variability in the true effect sizes across studies. The *Q*-statistic of 220.7281 (df = 6; *p* < 0.0001) decisively rejects the null hypothesis of homogeneity, while the *I*^2^ statistic of 98.36 supports this conclusion. This result indicates substantial true heterogeneity (98.36%) beyond mere sampling error, warranting the exploration of potential moderators.

### 3.4 Maximizing HOTS

Among the included studies, 18 effect sizes were extracted with reference to the impact of enrichment studies on HOTS of gifted students, ranging from 0.48 to 16.67. A large effect was found on the mean effect size (*g* = 3.55, 95% CI [1.93, 5.17]), indicating that enrichment interventions had a large effect on HOTS (Table 5).

**Table 5:** Effectiveness of enrichment programs on higher-order thinking skill development.

N	Study	Effect sizes	SE	Lower CI 95%	Upper CI 95%
1	Alshahri (2023)	0.84	0.09	0.25	1.44
2	Mnsoor and Harriry (2020)	6.32	0.20	5.45	7.20
3	Alkhuzaim and Al-Qutaim (2022)	2.22	0.13	1.52	2.93
4	Muammar (2022)	2.69	0.95	0.78	4.60
5	Al-Besher (2021)	1.39	0.17	0.60	2.19
6	Mohammad (2013)	5.11	0.09	4.53	5.68
7	Nassar (2013)	2.99	0.12	2.31	3.67
8	Alqapaly and Grwan (2009)	4.67	0.42	3.44	5.98
9	Alqapaly and Grwan (2009)	0.48	0.03	0.16	0.79
10	Mohammed (2010)	16.68	4.47	12.53	20.82
11	Al-Sayed et al. (2021)	5.18	0.54	3.73	6.62
12	Asimi (2023)	3.29	0.16	2.51	4.07
13	Abdulhalim et al. (2023)	8.77	1.93	6.05	11.50
14	Basser (2019)	2.687	0.951	0.78	4.60
15	Saad (2016)	−2.69	0.25	−3.67	−1.70
16	Almania (2024)	1.885	0.144	1.14	2.63
17	Jamali (2019)	1.393	0.166	0.60	2.19
18	Sabra et al. (2025)	0.844	0.093	0.25	1.44

The heterogeneity analysis revealed statistically significant variability in true effect sizes across studies. The *Q*-statistic of 548.1668 (df = 17; *p* < 0.0001) decisively rejects the null hypothesis of homogeneity, and the *I*^2^ statistic of 98.83 supports this conclusion. This result indicates a substantial degree of true heterogeneity (98.83%) beyond mere sampling error, warranting the exploration of potential moderators.

The following sections illustrate how grade levels and gender moderate the effects of enrichment programs in maximizing cognitive and affective abilities and HOTS.

### 3.5 Moderator analysis

#### 3.5.1 Regarding cognitive skills

Moderator analysis did not support the hypothesis that gender played a moderating role in the effects of enrichment programs on cognitive abilities [Qb (2) = 4.065, *p* > 0.05]. The effect size was greater for the combined population (male and females) (*g* = 3.27, *p* < 0.0001) than for only male or only female students (*g* male = 2.5, *p* = 0.0001; *g* female = 1.51, *p* = 0.032).

Moderator analysis also showed that grade level did not moderate the effect of enrichment programs on cognitive abilities [Qb (3) = 1.91, *p* = 0.5917]. The highest effect size was found in middle grade (*g* = 3.04, *p* = 0.0001), while the lowest was in secondary grade (*g* = 2.02, *p* = 0.0001) (Table 6).

**Table 6:** Effect sizes of enrichment programs on cognitive skill development, by group and total, across study moderators.

Mood	*K*	E. S	SE	95% CI		Q	Qb	I2
Gender				Lower	Upper		4.065	
Male	8	2.51	0.50	1.52	3.49	165.51		96%
Female	4	1.51	0.70	0.13	2.88	15.81		81%
Combined	8	3.27	0.53	2.23	4.31	108.03		94%
Grade Level							1.91	
Kindergarten	5	2.66	0.59	1.51	3.82	32.81		88%
Primary	4	2.59	0.63	1.36	3.82	69.06		96%
Middle	5	3.04	0.58	0.27	1.91	52.20		92%
Secondary	6	2.02	0.50	5.27	1.04	107.81		95%

#### 3.5.2 Regarding affective skills

Moderator analysis showed that gender did not play a moderating role in the effect of enrichment programs on affective skills [Qb (1) = 1.23 *p* > 0.05]. The effect size was greater for the combined population (male and females) (*g* = 6.28, *p* < 0.0001) than for male students (*g* = 3.72, *p* = 0.031).

Moderator analysis further showed that grade level did not play a moderating role in the effect of enrichment programs on affective skills [Qb (2) = 5.68, *p* = 0.0584]. The highest effect size was found in secondary grades (*g* = 9.22, *p* = 0.0001), while the lowest was in middle grades (*g* = 3.38, *p* = 0.04) (Table 7).

**Table 7:** Effect sizes of enrichment programs on affective skill development, by group and total, across study moderators.

Mood	*K*	E. S	SE	95% CI		*Q*	Qb	I2
Gender				Lower	Upper		1.23	
Male	3	3.72	1.73	0.33	7.12	96.6O		98%
Combined	4	6.28	1.53	3.29	7.41	79.43		96%
Grade Level							5.68	
Primary	2	3.97	1.96	0.13	7.81	12.18		92%
Middle	3	3.38	1.59	0.27	6.49	80.83		98%
Secondary	2	9.22	2.02	5.27	13.18	31.29		97%

#### 3.5.3 Regarding HOTS

Moderator analysis did not support hypothesis that gender played a moderating role in the effect of enrichment programs on HOTS [Qb (2) = 0.011, *p* > 0.05]. The effect size was greater for male students (g = 3.42, *p* < 0.0001) than for female students and the combined population (*g* female = 3.29, *p* = 0.0005; *g* combined = 3.41, *p* = 0.0051).

Table 8 shows that the effect size varied with grade level [Qb (2) = 29.5781, *p* < 0.0001]. The effect size was the greatest for kindergarten (*g* = 4.956, *p* = 0.002), while it was the lowest for secondary grades (*g* = 1.971, *p* = 0.0001).

**Table 8:** Effect sizes of enrichment programs on higher-order thinking skills, by group and total, across study moderators.

Mood	*K*	E. S	SE	95% CI		*Q*	Qb	I2
Gender				Lower	Upper		0.011	
Male	8	3.42	0.85	1.75	5.09	85.26		92%
Female	6	3.29	0.95	1.43	5.16	315.99		98%
Combined	4	3.41	1.22	1.03	5.79	145.02		98%
Grade Level							29.5781*	
Kindergarten	3	4.9558	0.54	3.89	6.02	13.05		85%
Primary	6	2.1729	0.15	1.36	1.87	158.22		97%
Middle	0	–	–	–	–	–		–
Secondary	9	1.9707	0.11	1.77	2.17	347.32		98%

*p < 0.05.

### 3.6 Publication bias

Publication bias was assessed for 45 effect sizes derived from 27 selected studies. Three methods were adopted to assess publication bias: funnel plot combined with Eigger regression analysis, Duval and Tweedie’s trim and fill, and Rosenthal’s fail-safe N. Figure 3 shows an asymmetric funnel plot, indicating potential publication bias. Regression analysis for funnel plot asymmetry produced a result of (*t* = 5.7195, df = 43, *p* < 0.0001). The number of studies to be added and the adjusted effect size were determined. The addition of 18 hypothetical missing studies was required to obtain a symmetric funnel plot. Rosenthal’s fail-safe N test found that the total number of missing studies that could have resulted in a *p*-value greater than alpha was 32,864, greater than the tolerance level reported by Rosenthal (1979, p. 10). The large fail-safe N sample size indicates that the current study appears to be free from the file-drawer problem.

**Figure 3:**
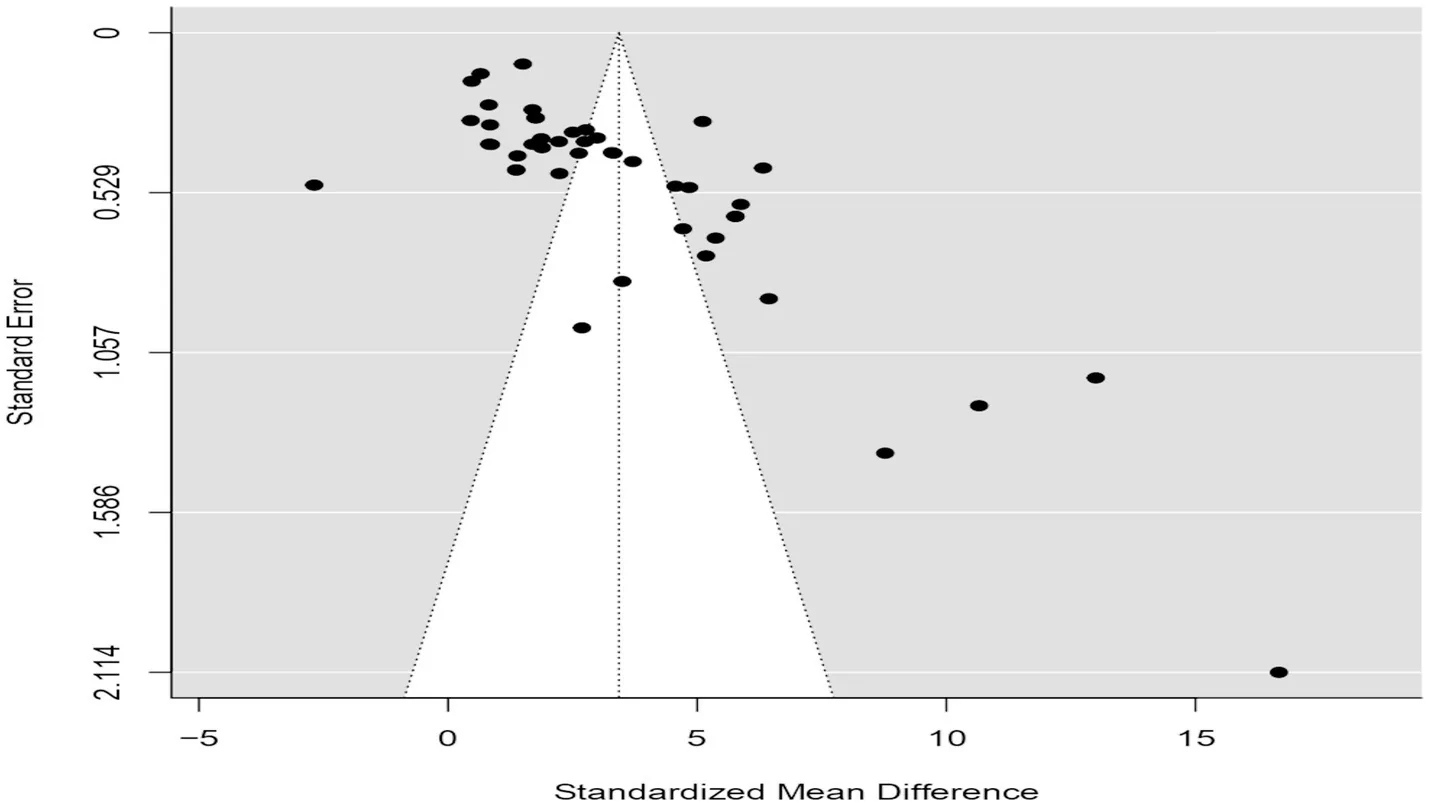
Funnel plot of standard error by Hedges’ *g*.

## 4 Discussion

The present meta-analysis investigated the effectiveness of enrichment programs in developing cognitive and affective abilities and HOTS in gifted students in Arab countries, investigated whether gender and grade level moderated these effects. A very large overall effect (*g* = 3.42) was observed, with large effects on cognitive (*g* = 2.73), affective (*g* = 5.21), and higher-order thinking outcomes (*g* = 3.55). Gender did not significantly moderate these effects, and grade level moderated higher-order thinking outcomes but not cognitive or affective outcomes. The substantial heterogeneity found across all domains indicates considerable variation among studies.

### 4.1 Overall effectiveness of enrichment programs

This study supports previous evidence that enrichment programs can promote the development of gifted learners. Kim (2016) reported positive effects on academic achievement (*g* = 0.96) and socioemotional development (*g* = 0.55), and Gül and Ayık (2025) found positive cognitive and affective outcomes. Thus, the findings of this study align with previous findings concerning the direction of the effect, but the magnitude observed here is considerably larger.

However, this does not imply that enrichment programs in Arab countries are more effective. Differences in study design, sample size, intervention characteristics, comparison conditions, and measurement methods could partly explain the larger estimates. The Arab educational context could also be relevant. Gifted education programs across Arab countries vary in terms of their identification procedures, program structures, curricula, and opportunities for advanced learning. Where regular classrooms provide fewer opportunities for differentiated or inquiry-based learning, structured enrichment could represent a more substantial change in students’ educational experiences. However, as contextual characteristics were not directly tested as moderators, this explanation remains tentative.

### 4.2 Cognitive outcomes

Enrichment programs had a large and positive effect on cognitive outcomes (*g* = 2.73), in line with Kim (2016), who reported an effect of *g* = 0.96 on academic achievement, and Gül and Ayık (2025), who found an effect of *g* = 1.14 on cognitive skills. The consistency of these results supports the cognitive benefits of enrichment, although the larger estimate found in the present study requires cautious interpretation.

Enrichment provides depth and complexity, inquiry, and advanced problem-solving, and this could help explain its positive cognitive effects. However, the cognitive outcomes synthesized here were diverse, ranging from academic achievement and scientific and mathematical concepts to language and other cognitive skills. Together with the methodological differences, this may partly explain both the large pooled effect and the very high heterogeneity (*I*^2^ = 98.24%).

### 4.3 Affective outcomes

The largest effect was in affective outcomes (*g* = 5.21). Although this result aligns with previous research that indicates positive non-cognitive effects, its magnitude is much greater than the socioemotional effect reported by Kim (2016; *g* = 0.55) and the affective-personal (*g* = 0.51) and social-affective (*g* = 0.44) effects reported by Gül and Ayık (2025). Thus, these studies agree only on the direction of the effect, not the magnitude.

One explanation for these considerations could lie in measurement, specifically self-report. Such measures could be sensitive to participants’ perceptions and expectations. Response-shift processes could also influence post-intervention ratings when participation changes students’ understanding of the construct being assessed (Anvari et al., 2023). The high heterogeneity (*I*^2^ = 98.36%) implies that the large affective effect should be interpreted cautiously.

### 4.4 Higher-order thinking outcomes

Enrichment programs had a large effect on HOTS (*g* = 3.55), including creativity, critical thinking, problem-solving, and decision-making. This finding is consistent with previous evidence that enrichment can foster advanced cognitive processes; furthermore, it extends this evidence by examining higher-order thinking as a distinct outcome domain in Arab educational contexts.

This finding accords with the nature of enrichment programs, as they commonly emphasize open-ended inquiry, complex problem-solving, creative production, and research activities. However, the high heterogeneity (*I*^2^ = 98.83%) suggests that effects can vary substantially across programs. Programs explicitly targeting creativity or problem-solving could produce different outcomes from programs where these skills are secondary objectives.

### 4.5 Moderating the roles of gender and grade level

Gender did not significantly moderate the cognitive, affective, or higher-order thinking outcomes. While subgroup effect sizes varied descriptively, the differences were not statistically significant. Thus, enrichment could provide a broadly comparable range of benefits for gifted male and female students. However, some gender categories included relatively few effect sizes, and several studies used combined-gender samples; therefore, the absence of significant moderation should not be interpreted to represent evidence of complete gender equivalence.

Grade level did not significantly moderate cognitive or affective outcomes but did significantly moderate higher-order thinking outcomes. This differs from Kim (2016), who found grade-level differences in both academic and socioemotional effects. These and other differences could reflect variations in terms of educational contexts, program structures, outcome classifications, or samples.

For higher-order thinking, the largest effect was found at the kindergarten level, and the smallest was at the secondary level. Younger students could have greater scope for observable development in their emerging HOTS, while enrichment programs at different educational stages could also differ. However, as some grade-level subgroups included relatively few studies, this finding requires replication and is not in itself evidence of a general developmental pattern.

### 4.6 Heterogeneity and robustness of the findings

The exceptionally high level of heterogeneity is important for interpreting the findings. The overall *I*^2^ reached approximately 99%, with similarly high values across the three outcome domains. This variation is most likely related to differences in program content, duration, intensity, delivery, sample, research design, comparison condition, and measurement instruments. Consequently, the pooled effects are averaged from diverse studies and do not represent effects that should be expected from every enrichment program.

This heterogeneity could also help explain why the present estimates are larger than those reported by Kim (2016) and Gül and Ayık (2025). Instead of indicating uniformly stronger effects in Arab countries, the findings show considerable variability in the Arab evidence base. Future research should therefore examine additional moderators.

Findings regarding publication bias also require a balanced interpretation. Funnel plot asymmetry and the significant Egger test suggest small-study or publication-related effects, and the large fail-safe N indicates that many null findings would be required to eliminate the statistical significance of the overall effect. Because a large fail-safe N does not rule out publication bias, the positive direction of the findings appears to be robust; however, the magnitude of the pooled effects should be interpreted with caution.

### 4.7 Theoretical and practical implications

This study is consistent with previous research, finding positive cognitive and affective effects of enrichment programs, and extends the literature to include higher-order thinking outcomes in Arab educational contexts. The large effect sizes obtained in this analysis indicate that enrichment should not be viewed as a uniform intervention. Its effectiveness depends on program design, objectives, instructional strategies, duration, student characteristics, and measures used to evaluate outcomes.

The findings support continued investment in enrichment opportunities for gifted learners, emphasizing the need for stronger program evaluation. Future studies should use rigorous experimental or quasi-experimental designs, adequate samples, validated measures, and detailed descriptions of interventions. Additionally, which enrichment approaches work best for which gifted learners, at which developmental stages, and under what educational conditions should be examined.

## 5 Conclusion

The findings of this meta-analysis demonstrate that enrichment programs can make a valuable contribution to the development of gifted students in Arab countries. Positive effects were found across cognitive and affective outcomes, as well as higher-order thinking skills, suggesting that the value of enrichment is not limited to academic development. The findings also showed that gender did not significantly account for differences in program effects. However, grade level was associated with differences in higher-order thinking outcomes but not with cognitive or affective outcomes.

At the same time, the large effects found in this study must be interpreted carefully. There was considerable variation across the included studies, and there were also indications of possible publication or small-study effects. Therefore, it would be misleading to assume that all enrichment programs produce effects of the same magnitude. Differences in program content, duration, implementation, participants, research design, and outcome measures may all contribute to the variation observed across studies.

Overall, the findings provide support for the continued use and development of enrichment programs for gifted students in Arab educational settings. However, they also point to the need for stronger evidence regarding how these programs work. Future studies should use more rigorous designs and well-validated measures and provide clearer descriptions of program characteristics and implementation. Further research is particularly necessary to determine which types of enrichment are most beneficial, for which gifted students, and under what educational conditions.

### 5.1 Limitations

- High heterogeneity limits generalizability.
- Variations in design and measurement could inflate estimates.
- Possible indicators of publication bias were observed despite a large fail-safe N.
- Small subgroup sizes and absence of key moderators (e.g., program type, dosage, country) were noted.

### 5.2 Implications

- Institutionalize enrichment programs, linking them to clear performance standards.
- Balance cognitive, affective, and higher-order thinking components, with particular attention to secondary education.
- Employ standardized instruments and expand performance-based assessments.
- Train teachers to integrate higher-order thinking within the regular curriculum.

### 5.3 Future research directions

- Detailed moderator analyses should be conducted (including analyses by program type, dosage, design, measurement tools, and context).
- More rigorous experimental studies should be conducted, with larger samples and reporting that is more aligned with PRISMA guidelines.
- Each domain should be disaggregated into more specific indicators (e.g., achievement vs. self-regulated learning; motivation vs. self-concept; and creativity against critical thinking).

## Data Availability

The original contributions presented in the study are included in the article/supplementary material, further inquiries can be directed to the corresponding author.
